# Advancing Nurse‐Midwifery Education: A Quality Improvement Initiative for Competency‐Based Intrapartum Skills Laboratories

**DOI:** 10.1111/jmwh.70029

**Published:** 2025-09-20

**Authors:** Deanna Womack, Melissa Stec

**Affiliations:** ^1^ Nell Hodgson School of Nursing Emory University Atlanta Georgia; ^2^ School of Medicine Vanderbilt University Nashville Tennessee

**Keywords:** midwifery education, quality improvement

## Abstract

**Introduction:**

Maternal morbidity and mortality rates in the United States and Georgia remain alarmingly high, exceeding those of many low‐resource regions despite extensive interventions. Research highlights the role of competent midwifery care in addressing key contributing factors, such as limited health care access, insufficient prenatal care, and adverse social determinants of health. To expand the pool of qualified certified midwives and certified nurse‐midwives, there is a pressing need for robust midwifery education programs, reliable and valid evaluation tools for student assessment, and documentation of skill development and confidence improvement among trainees.

**Process:**

To evaluate preparedness and competency, a quality improvement project was initiated to assess the effectiveness and efficiency of the intrapartum simulation laboratory. A modified version of the National League for Nursing Student Satisfaction and Self‐Confidence in Learning tool was used. Third‐semester midwifery students at Emory University School of Nursing in Atlanta, Georgia, completed pre‐ and postlaboratory surveys, which included a Likert scale to measure confidence in the simulation laboratory's ability to meet their educational needs. Qualitative questions were incorporated to identify suggestions for laboratory improvements.

**Outcomes:**

Statistically significant improvements were observed in midwifery students’ pre‐ and postlaboratory assessments, particularly in their confidence regarding the skills reviewed, the alignment of simulation and laboratory time with their learning styles, and their trust in faculty members’ ability to effectively teach essential midwifery practices.

**Discussion:**

The findings validate the effectiveness of intentional teaching strategies and innovative simulation technologies in enhancing midwifery education. Increasing the number of competent midwives in practice represents a critical step in addressing the persistently high maternal morbidity and mortality rates in the United States. These teaching approaches and technologies can also be applied to other midwifery simulation laboratories and adapted for use in other advanced practice registered nursing specialties.

## INTRODUCTION

Maternal morbidity and mortality remain a critical challenge in the United States, with rates surpassing those of many low‐resource countries. Georgia has one of the leading US maternal death rates of 33.9 deaths per 100,000 live births (between 2018 and 2021).^1^ This rate increases to 66.6 and 90 deaths per 100,000 live births for Black women and women aged 35 to 44, respectively.^2^ In 2020, 861 women died in the United States of causes related to pregnancy and childbirth, leading to a maternal mortality rate of 23.8 per 100,000 live births.^1^ As seen in other high‐resource countries, many of these deaths could be prevented through access to well‐trained midwives, particularly for conditions like infection and hemorrhage.^3^

  

Continuing education (CE) is available for this article. To obtain CE online, please visit http://www.jmwhce.org. A CE form that includes the test questions is available in the print edition of this issue.

Access to care is a key driver of Georgia's grim statistics. Of the 82 Primary Care Service Areas outside metro Atlanta, 52% lack adequate obstetric providers.^4^ Limited access correlates with increased risks of preterm birth, low birth weight, and poor maternal outcomes. Social determinants of health, including disparities in prenatal care quality, poverty, chronic stress, and systemic racism, exacerbate maternal health inequities, particularly for Black women.^5^ The Georgia Maternal & Infant Health Research Group conducted a survey assessing Georgia's obstetric care and access crisis and the utility of midwifery as a solution. The results identified 3 key factors: (1) more midwifery students intended to practice in rural Georgia than other surveyed obstetric providers, (2) cost‐effective solutions should include certified nurse‐midwives (CNMs), and (3) CNMs can address disparities through innovative practices and enhanced reimbursement models.^4^ Research has confirmed that care provided by CNMs and certified midwives (CMs) improves maternal and neonatal outcomes, is cost‐effective, and is as safe, if not safer, than physician‐led care.^6^, ^7^ Although CMs are not currently licensed in Georgia, developing robust midwifery programs to graduate competent CMs and CNMs is a vital step toward reducing maternal mortality and morbidity in Georgia and nationwide.

Currently in Georgia, there remains only 2 onsite nurse‐midwifery programs: one at Emory University and the other at Georgia College & State University. Several other out‐of‐state online programs, such as Frontier, Georgetown, and University of Cincinnati, exist for students, with in‐state programs graduating less than 35 students annually. According to the American Midwifery Certification Board 2025 data, there are 633 CNMs living in Georgia.^8^ This number has steadily increased over the last 5 years and likely represents a growing midwifery workforce.^9^ In Georgia, CNMs are licensed by the State Board of Nursing as advanced practice registered nurses (APRNs). In 2022, CNMs attended 21,077 births in Georgia or 34.6 births per midwife, with 25% of all vaginal births being attended by midwives.^10^

**QUICK POINTS**

✦ Simulation and skills laboratories are critical for preparing midwifery students for clinical experiences by bridging theoretical knowledge and hands‐on practice.
✦ This quality improvement project implemented multimodal learning for students while using the National League for Nursing tool to measure confidence and satisfaction.
✦ Responses demonstrated statistically significant improvements in midwifery students’ confidence regarding key skills, particularly in the context of simulation‐based learning.
✦ Intentional teaching strategies and the use of innovative simulation technologies foster student development, emphasizing the potential to improve midwifery education outcomes.

### Background

At Emory University School of Nursing, the nurse‐midwifery specialty is offered in a hybrid format and includes 15 to 25 students per cohort. Students have the option for either a master of science in nursing or doctor of nursing practice degree at program completion. This project involves third‐semester midwifery students who participate in a 5‐day intrapartum laboratory intensive, designed to prepare them for clinical experiences. Nurse‐midwifery students begin intrapartum clinicals in their third semester, following an intensive hands‐on laboratory experience designed to demonstrate their foundational knowledge and clinical skills. This laboratory combines content reviews, skill demonstrations, and simulation activities. Students practice in small groups through various roles, including midwife, triage nurse, and labor nurse, during simulations of both routine and complex births, such as precipitous births, nuchal cord management, shoulder dystocia, postpartum hemorrhage, and normal outcomes.

The laboratory previously operated in a single large classroom with 18 to 20 students rotating through stations. Faculty observed, supported, and provided feedback during the first 4 days of ungraded activities. The final day included 2 summative assessments: an online content examination and an objective structured clinical examination (OSCE) using a low‐fidelity model. Although this structure reinforced prior didactic content, it relied on faculty demonstrations, low‐fidelity simulations, and individual student practice.

Student feedback from previous laboratory surveys identified several areas for improvement, including more faculty support during suturing practice, clearer activity expectations, additional birth models for simulation, and increased content on fetal monitoring, labor management, and postpartum complications. Students also suggested smaller group sizes for simulations, repeated practice of breech and malpresentation scenarios, and expanded faculty availability. Despite its strengths, the laboratory had significant gaps, such as limited use of valid and reliable evaluation tools, inadequate documentation of students’ skill improvement and confidence, and a lack of structured simulation prebriefs and debriefs. Additionally, recent faculty turnover disrupted continuity, and there was no formal process for postlaboratory feedback from students or debrief meetings among faculty.

This intrapartum laboratory is critical for preparing midwifery students for clinical experiences by bridging theoretical knowledge and hands‐on practice. However, improvements were needed to address identified gaps and enhance its effectiveness. Incorporating valid evaluation tools, structured simulation briefings, comprehensive feedback mechanisms, and additional faculty resources could improve student outcomes. Enhancing the intrapartum laboratory was therefore an essential step in preparing competent midwives who could contribute to reducing disparities in Georgia (and elsewhere) while ensuring high‐quality, cost‐effective care.

This quality improvement (QI) project aimed to evaluate the effectiveness and efficiency of an intrapartum laboratory after integrating evidence‐based teaching strategies, innovative technology use, and assessments of midwifery student confidence both before and after the laboratory, alongside summative measures of student competency.

### Literature Review

Nurse‐midwifery is a competency‐based APRN specialty in the United States. The integration of competencies from the American College of Nurse‐Midwives (ACNM) and the International Confederation of Midwives (ICM) helps to define the performance objectives for midwifery programs in the United States. Both organizations’ definitions of competency overlap, with a focus on knowledge and skills. However, ACNM emphasizes the importance of skills, whereas ICM incorporates professional behaviors as a third component necessary for a midwife to be considered competent.^11^, ^12^ Although most scholars agree that competency is a combination of theoretical knowledge, practical skills, and the demonstration of specific behaviors,^3^, ^11^, ^12^, ^13^, ^14^ measuring competency remains an elusive challenge.^3^, ^13^

The components of competency‐based education (CBE) in midwifery programs vary significantly across institutions. Jones et al argued that personal characteristics and traits of students form the foundation for learning outcomes, with experience facilitating the acquisition of skills, knowledge, and abilities.^15^ The integration of these experiences eventually leads to competency, which is assessed through performance demonstrations. Kraus et al expand upon this framework, asserting that demonstrating defined skills and learning outcomes is central to CBE.^16^ This perspective aligns with the learning theories of Dreyfus and Benner, who argued that it is the combination of theoretical knowledge and practical experience that leads to competence in any field.^17^, ^18^ Extensive research in academia and practice has focused on refining CBE frameworks. Kraus et al highlight the necessity of not only defining core competencies but also measuring students’ knowledge, skills, and abilities effectively.^16^

Many scholars agree on the need for reliable and valid tools to assess student competency.^3^, ^12^, ^13^, ^14^, ^15^, ^16^, ^17^, ^18^, ^19^ Some suggest that standardized assessment tools could help measure competency across various programs.^13^, ^14^, ^19^ Woeber further clarifies that student progression and preparedness are often not directly linked to the intended outcomes of learning or clinical competencies, pointing to a gap in accurate assessments.^13^ In light of limited clinical sites and diverse educational needs, simulation‐based learning has become an integral component of prelicensure and graduate education for nurses, APRNs, and CNM/CMs. Simulation offers a controlled environment that allows students to build confidence, demonstrate skills, and enhance their critical thinking and decision‐making abilities.^3^, ^20^, ^21^ Through simulation, students can gain and demonstrate competency in various clinical skills.^20^, ^22^

In midwifery education, simulation is particularly useful for developing skills in managing perinatal emergencies. Although not every student will encounter emergencies such as cord prolapse, shoulder dystocia, or postpartum hemorrhage during clinical rotations, all students can practice and hone their management skills through simulation.^19^, ^22^, ^23^ Simulation provides a safe learning space where students can make mistakes, reflect on their experiences, and integrate their clinical skills.^3^, ^19^, ^22^ The International Nursing Association for Clinical Simulation Learning has established benchmarks for simulation processes.^24^ Research indicates that the debriefing phase of simulation is crucial for learning, as it fosters reflection that integrates knowledge, improves self‐awareness, promotes safe patient care, and enhances teamwork.^20^, ^23^, ^24^ This reflective process helps reinforce critical thinking, allowing students to explore alternative actions when initial decisions do not yield optimal outcomes.^23^, ^24^

The foundation for the student surveys used in this project was a modified version of the National League for Nursing (NLN) Student Satisfaction and Self‐Confidence in Learning (SSSCL) tool, a well‐established instrument that has been widely employed in nursing education since its development in 2006.^25^ This tool is part of the NLN's broader suite of validated evaluation instruments designed to assess the effectiveness of simulation‐based education and learner outcomes. The validity and reliability of the SSSCL tool has been extensively studied and affirmed in the literature. Notably, Franklin et al conducted psychometric testing using a sample of prelicensure novice nurses to evaluate the internal consistency and construct validity of the tool.^26^ Their results confirmed that the instrument reliably measures both student satisfaction with learning experiences and their self‐confidence in learning through simulation. These 2 dimensions are critical indicators of the educational impact of simulation‐based learning in health care education.

Furthermore, this tool has demonstrated relevance and applicability in specialized clinical education settings, including midwifery simulation training. Andrighetti et al successfully implemented the SSSCL tool in a study involving simulations of high‐risk perinatal emergencies, such as shoulder dystocia and postpartum hemorrhage.^27^ Their findings highlighted an increase in student confidence and perceived preparedness when managing these complex clinical scenarios, outcomes closely aligned with the goals of this project. Given its strong psychometric properties and history of use in similar contexts, the modified NLN SSSCL tool serves as an appropriate and evidence‐based method for evaluating student perceptions in this project. Its use supports the ongoing aim to enhance educational experiences, foster learner confidence, and ensure that simulation‐based instruction remains a rigorous and reflective component of health care training.

## PROCESS

The project was submitted to university institutional review board and was determined not to involve human participants research, as it was classified as a QI initiative. Multiple prelaboratory faculty meetings were held to finalize the content, supplies, setup, teaching strategies, simulation modalities, tool development, and new assessment methods. The Alternative Assessment framework was employed to address diverse learning styles, offering multiple opportunities for students to demonstrate their content integration and critical thinking skills through both traditional and innovative formats. The Substitution, Augmentation, Modification and Redefinition model was used to optimize current technology and incorporate new modalities, enriching the educational experience.

The project involved 18 third‐semester nurse‐midwifery students, most of whom entered the program directly after completing an accelerated prelicensure nursing program. Students came from diverse academic backgrounds, most holding degrees in social or biological sciences, with a range of life and work experiences. The faculty team consisted of 4 experienced CNMs, including the program director, course coordinator, and laboratory coordinator, along with guest presenters from a nearby birth center and a doula. Three laboratory staff members provided technological support.

This laboratory took place in a large classroom with additional smaller rooms for group work, faculty demonstrations, and birth simulations. Students participated in both individual activities at their tables and group rotations in various areas for simulations and content coverage led by faculty.

Initially, assimilation of content was not formally assessed over the first 4 days of the laboratory, although individual feedback was provided as students reviewed material, refined their skills, and engaged in various simulations. Two summative evaluations were implemented: an online intrapartum knowledge assessment (Supporting information: Table S1) administered before the laboratory intensives, and a midfidelity birth OSCE (Supporting information: Table S2) conducted on the fourth day. Students scoring below 90% on either the quiz or OSCE were provided with remediation and additional faculty support, followed by a reevaluation the following week.

The Knowledge to Action (KTA) framework guided the intervention's implementation, incorporating 9 innovative teaching strategies in addition to established methods.^28^ These strategies were informed by surveys from the previous student cohort. Prelaboratory mini‐lectures were delivered to review key didactic content and set clear performance expectations for each session. Prelaboratory preparation materials were enhanced to target information crucial for student success, and faculty resources were increased to provide clearer, more robust feedback. Opportunities for faculty‐led skill demonstrations, as well as simulation prebriefs and debriefs, were expanded. During the laboratory, enhanced simulation scenarios were employed, alongside increased structure and technology for skills practice. Technology played a key role, including live‐feed suture practice streamed to multiple screens and the use of advanced tools and instructional videos. Planned experiences included amniotomy, internal monitor placement, suturing with realistic materials, and both mid‐ and high‐fidelity simulations. The assessment component was redesigned to improve the OSCE, with faculty prelaboratory training to ensure evaluation consistency. Students were required to complete self‐reflective notes, along with pre‐ and postlaboratory surveys on content, confidence, satisfaction, and faculty proficiency.

Student surveys included a modified version of a validated NLN tool to assess student confidence and satisfaction (Supporting information: Tables S3 and S4). The study used an observational, one‐group posttest design to document outcomes following the 4‐day laboratory intensive with all 18 third‐semester midwifery students. A pre‐ and posttest student self‐evaluation was administered via a 5‐question Likert scale, with an additional qualitative question regarding suggested laboratory improvements (Supporting information: Tables S5 and S6). The final component of the evaluation involved a one‐group posttest design with skills checklists for the OSCE summative evaluation on the fourth day. Pre‐ and posttest surveys were matched and de‐identified to ensure participant anonymity.

The effectiveness of the enhanced teaching strategies for the laboratory intensives was assessed through daily faculty observations, debrief discussions following each laboratory day, and student surveys. After each laboratory day, faculty gathered for debriefing and to confirm plans for the following day. A comprehensive faculty debrief took place 2 weeks after the laboratory, which included all faculty and laboratory staff, followed by a final debrief a month later to discuss the results and initial plans for future laboratory iterations.

The project was developed in accordance with the SQUIRE (Standards for Quality Improvement Reporting Excellence) guidelines to ensure a comprehensive and transparent presentation of the methodology, interventions, and outcomes.

## OUTCOMES

Paired *t* tests were conducted to assess whether a significant difference existed between pre‐ and posttest survey results regarding students’ confidence and satisfaction with the midwifery intrapartum laboratory intensive. As illustrated in Table 1, statistically significant improvements were observed in students’ confidence levels concerning the skills covered during the laboratory. Students reported that simulation and practice time were beneficial to their learning, that the laboratory environment was well suited to their learning styles, and that faculty were effectively equipped to teach the necessary skills for midwifery practice. The last 6 items only appeared on the posttest (Table 2). The mean score for these items was between 4.5 and 5 on a 5‐point Likert scale.

**Table 1:** Pre/Posttest Comparison (N = 18)

Question	Pretest mean (SD)^a^	Posttest mean (SD)	*P* Value
I feel confident, prelaboratory, about the skills that will be reviewed.	2.6 (1.04)	4.3 (0.49)	<.001
I feel that simulation and practice time in the laboratory will benefit my learning.	4.7 (0.65)	4.9 (0.24)	.31
I feel that simulation and laboratory time is well suited to my learning style.	4.3 (0.92)	4.4 (0.24)	.01
I am confident that my faculty are well prepared to teach me skills necessary for midwifery practice.	4.4 (0.70)	5 (0)	0.002
The simulation provided me with a variety of learning materials and activities to promote my learning the midwifery curriculum.	N/A	4.9 (0.24)	N/A^a^
I am confident that I am mastering the content of the simulation and laboratory activities that my instructors presented to me.	N/A	4.5 (0.62)	N/A^a^
I am confident that I am developing the skills and obtaining the required knowledge from this simulation/laboratory intensive to perform necessary tasks in a clinical setting.	N/A	4.5 (0.62)	N/A^a^
My instructors used helpful resources to teach the simulation/laboratory intensive.	N/A	4.9 (0)	N/A^a^
I know how to use simulation and laboratory activities to learn critical aspects of these skills.	N/A	4.8 (0.43)	N/A^a^
I know how to get help when I do not understand the concepts covered in the simulation/laboratory.	N/A	4.9 (0.24)	N/A^a^

Abbreviation: NA, not applicable.

a 5‐point Likert scale with anchors 1 = strongly disagree and 5 = strongly agree.

**Table 2:** Questions Which Only Appeared in Posttest

Question	Circle Response^a^
The simulation provided me with a variety of learning materials and activities to promote my learning of the midwifery curriculum.	1 2 3 4 5
I am confident that I am mastering the content of the simulation and laboratory activities that my instructors presented to me.	1 2 3 4 5
I am confident that I am developing the skills and obtaining the required knowledge from this simulation/laboratory intensive to perform necessary tasks in a clinical setting.	1 2 3 4 5
My instructors used helpful resources to teach the simulation/laboratory intensive.	1 2 3 4 5
I know how to use simulation and laboratory activities to learn critical aspects of these skills.	1 2 3 4 5
I know how to get help when I do not understand the concepts covered in the simulation/laboratory.	1 2 3 4 5

^a^ 5‐point Likert scale with anchors 1 = strongly disagree and 5 = strongly agree

Table 3 presents the most frequent posttest responses regarding additional content or skills that would have been beneficial to include in the laboratory. Students indicated suturing and hand maneuvers were the most critical skills for inclusion in the laboratory. Students also stated more practice with normal birth from start to finish and exposure to various birth positions would have benefitted their learning and confidence.

**Table 3:** Prelaboratory Question: What Skills Would You Find Beneficial to Incorporate Into the Laboratory?

Response	Frequency (N = 18) n (%)
Suturing	8 (44)
Hand maneuvers	5 (27)
Postpartum hemorrhage drill	4 (22)
Shoulder dystocia drill	3 (17)
Reading and management of fetal monitor strips	2 (11)
More hands‐on practice time	2 (11)
More skill repetition practice time	2 (11)
Practice emergency scenarios	2 (11)
Practice different birth positions	2 (11)

When asked what faculty could do differently to improve their intrapartum laboratory intensive experience, students reported that the content on the first day was too slow, whereas the following days felt rushed (Table 4). Students also made suggestions about restructuring supplementary content and increasing practice time with more opportunity for repetition of skills (Table 5).

**Table 4:** Postlaboratory Question: What Additional Content/Skills Would You Find Beneficial to incorporate Into the Laboratory?

Response	Frequency (N = 18) n (%)
More normal birth practice from start to finish	6 (33)
Different birth positions	4 (22)
More pelvimetry	3 (17)
More practice time for skills	2 (11)
Scenario practice from start to finish	2 (11)

**Table 5:** Postlaboratory Question: What Can Faculty Do Differently to Improve Your Intrapartum Laboratory Intensives Experience?

Response	Frequency (N = 18) n (%)
First day was too slow	3 (17)
Days 2 through 4 felt rushed	2 (11)
For the doula's presentation, consider a shorter Zoom call and how midwives interact with doulas	22 (11)
More repetition of skills	2 (11)
More practice time	2 (11)
Increase laboratory time to more than one week	2 (11)
More normal birth practice	2 (11)
Have no other course in the middle of a laboratory day	2 (11)

## DISCUSSION

Simulation‐based laboratories are integral components of CBE in midwifery programs.^14^, ^19^, ^22^ This QI project demonstrated that integrating evidence‐based teaching strategies and technological innovations significantly enhanced nurse‐midwifery student confidence and satisfaction with an intrapartum laboratory intensive. The student surveys conducted before and after the laboratory intensives provided statistically significant evidence that laboratory time was beneficial and matched students’ learning styles, and that faculty were well prepared to teach essential skills. The findings align with previous studies that emphasize the value of simulation learning in improving student confidence, competence, critical thinking, decision‐making, and the management of infrequent obstetric emergencies.^3^, ^19^, ^20^, ^21^, ^22^, ^23^

The project measured substantial increases in students’ perceived knowledge acquisition and skill demonstration, both of which are key components of competency as identified by various nursing and midwifery experts.^3^, ^11^, ^12^, ^13^, ^20^ Several of the new teaching strategies implemented in this laboratory were highlighted by Fullerton et al as essential to CBE.^3^ Although the measurement of competency remains inconsistent, McMahon et al and Woeber support the use of valid and reliable assessment tools to evaluate student competence.^13^, ^19^ This project used an adaptation of the proven and reliable Student Satisfaction and Confidence Survey, developed by the NLN.^25^

### Strengths and Limitations

This project was limited by its small sample size and its focus solely on intrapartum content, which restricts the generalizability of the results. Future research could expand the sample size and explore a broader range of midwifery education content. Given the challenges in securing graduate‐level clinical preceptors, the results from this project may be helpful in the recruitment and retention of preceptors, especially for students with limited labor and birth experience. Demonstrating students’ competency at the beginning of clinical experiences could improve preceptor confidence in mentoring these students. The goal moving forward is to integrate these effective teaching strategies and technological innovations into all future midwifery laboratories while also exploring additional proven teaching, evaluation, and technological approaches.

It is important to note that suggestions were gathered from previous cohorts of students prior to this project. These survey recommendations informed the design of the current project, and many were incorporated into the QI laboratory intensive. Comments that could not be addressed in this cohort's laboratory were either integrated into the following semester's laboratories or were not feasible due to ongoing COVID‐19 restrictions. In response to student earlier feedback, faculty increased opportunities for practicing normal births, different birth positions, shoulder dystocia drills, and postpartum hemorrhage drills in the subsequent semester. Actively involving students in the process and informing them how their feedback has shaped the curriculum will further improve student morale and satisfaction.

This project addressed several key gaps, including the need for valid, reliable student evaluation tools, the documentation of student progress in confidence and skills, and specific laboratory deficiencies such as faculty evaluations and the integration of simulation prebriefing and debriefing sessions. Additionally, faculty held debrief meetings to review what worked and to plan for future laboratory sessions. The anticipated impact of these findings could lead to improvements in midwifery student competence, laboratory efficiency, and overall student satisfaction.

## CONCLUSION

This QI project demonstrated the positive impact of evidence‐based teaching strategies and technological innovations on nurse‐midwifery students’ confidence, satisfaction, and competency in an intrapartum laboratory intensive. The use of simulation and hands‐on learning, coupled with pre‐ and postlaboratory assessments, significantly enhanced students’ perceived knowledge acquisition and skill development. These findings align with previous research emphasizing the value of simulation in midwifery education, particularly in improving clinical skills and decision‐making in obstetric emergencies.

Through faculty collaboration and thoughtful integration of feedback from prior student cohorts, this project addressed key areas for improvement in laboratory design, content delivery, and student assessment. Notable enhancements included increased simulation scenarios, more skill practice opportunities, and tailored instructional strategies. Additionally, the incorporation of the KTA framework ensured that teaching strategies were continuously refined based on student input, promoting an adaptive and responsive learning environment.

Future research could expand the sample size and include a broader range of midwifery topics, with potential applications to other advanced practice midwifery and nursing programs. Additionally, findings from this project can inform future preceptor recruitment and retention efforts, particularly for students lacking prior labor and birth experience.

Ultimately, this QI project contributes to the advancement of midwifery education by improving measurement of clinical competence, fostering student confidence, and addressing gaps in current training practices. Through ongoing refinement of teaching strategies and assessment tools, the project has the potential to positively impact the future of nurse‐midwifery education and contribute to improving maternal care outcomes by cultivating a skilled and confident workforce.

## CONFLICT OF INTEREST

The authors have no conflicts of interest to disclose.

## Supporting information




**Table S1**. IP Knowledge Quiz

**Table S2**. Intrapartum Normal Birth OSCE Rubric and Instructions

**Table S3**. Prelab Student Assessment Survey

**Table S4**. Post‐laboratory Student Assessment Survey

**Table S5**. Pre‐Laboratory Intensive Assessment

**Table S6**. Post‐Laboratory Intensive Assessment

**Materials S1**. Revised Standards for Quality Improvement Reporting Excellence (SQUIRE 2.0) September 15, 2015
