# Supplementary material for: Advancing Nurse‐Midwifery Education: A Quality Improvement Initiative for Competency‐Based Intrapartum Skills Laboratories
Source: J Midwifery Womens Health. 2025 Sep 20;71(2):283–9. doi: 10.1111/jmwh.70029 (PMC13067923; doi:10.1111/jmwh.70029)
Supplement: Supplementary file 1 — Table S1. IP Knowledge Quiz [file JMWH-71-283-s005.docx]

**Table S1. IP Knowledge Quiz**

**Labor Quiz**

**Question 1**

Active management of placental delivery involves controlled cord traction following signs of placental separation.

- True
- False

**Question 2**

Which of the following is a sign of placental separation?

- Gush of blood from the vagina
- Increased comfort of the mother
- Uterus becomes boggy
- Uterus descends into the abdomen

**Question 3**

Estimation of blood loss by the CNM is a very reliable measure of blood loss.

- True
- False

**Question 4**

Which of the following is the biggest risk factor for placenta accreta?

- Length of second stage
- Maternal age
- Uterine rupture
- Cesarean

**Question 5**

Which of the following is NOT a strategy associated with AMTSL (active management of the third stage of labor)?

- Uterine massage after delivery of the placenta
- Immediate cord clamping
- Controlled cord traction
- Pitocin administered after delivery of the baby's anterior shoulder

**Question 6**

The umbilical cord should have:

- 1 artery and 1 vein
- 1 vein and 2 arteries
- 2 arteries and 1 vein
- 2 veins and 1 artery
- None of the above

**Question 7**

The diagnosis of retained placenta is made when the placenta has not been expelled within ___ minutes of infant’s birth.

- 15 minutes
- 45 minutes
- 30 minutes
- 60 minutes

**Question 8**

In the __________ mechanism, the placenta separates centrally. The fetal side of the placenta—the side with a smooth, shining membrane continuous with the sheath of the chorion (which also covers the umbilical cord)—drops to the lower portion of the uterus, and then exits through the cervix into the vagina and onto the woman’s pelvic floor.

**Question 9**

Which medication is first line in the treatment of postpartum hemorrhage?

- Cytotec
- Hemobate
- Methergine
- Oxytocin

**Question 10**

Which of the following is not potential complication resulting from active management of the placenta?

- Postpartum hemorrhage
- Retained placenta
- Avulsed cord
- Uterine inversion
