# Supplementary material for: Advancing Nurse‐Midwifery Education: A Quality Improvement Initiative for Competency‐Based Intrapartum Skills Laboratories
Source: J Midwifery Womens Health. 2025 Sep 20;71(2):283–9. doi: 10.1111/jmwh.70029 (PMC13067923; doi:10.1111/jmwh.70029)
Supplement: Supplementary file 2 — Table S2. Intrapartum Normal Birth OSCE Rubric and Instructions [file JMWH-71-283-s004.docx]

| **Table S2. Intrapartum Normal Birth OSCE Rubric and Instructions** | | | | |
| --- | --- | --- | --- | --- |
| **Situation** | **Techniques** | **Points** | | **Comments** |
| Assess vital signs | Review chart and EFM strip before seeing patient |  | 4 |  |
| Assess EFM strip |  |  |  |  |
| Assess patient history and birth plan (on chart) |  |  |  |  |
| Handwashing or foam in |  |  |  |  |
| **2^nd^ stage** | | | | |
| Introduction: name and purpose | Verbalize upon meeting patient |  | 5 |  |
| Don sterile gown and gloves |  |  |  |  |
| Prepare table and patient |  |  |  |  |
| Identify fetal presentation and position | Verify vertex and position |  | 2 |  |
| Control the speed of delivery | Use hand to keep head flexed  Coach pushing appropriately |  | 2 |  |
| Take measures to prevent/minimize tearing | Positioning to reduce stress on perineum  Strategy(ies) to decrease friction |  | 2 |  |
| Check for and manage nuchal cord | After delivery of head, before/ during delivery of body |  | 2 |  |
| Manage delivery of the shoulders and body | Keep arms near baby’s body  Baby to maternal abdomen |  | 2 |  |
| Ensure baby’s effective transition | Assess flexion, breathing, color, heart rate, and response  Skin to skin, warm blanket |  | 2 |  |
| **3^rd^ stage** |  |  |  |  |
| Order oxytocin | Communicate appropriately with RN  Give specific order |  | 2 |  |
| Clamp and cut cord | Verbalize when this should happen  Perform technique |  | 2 |  |
| Collect cord blood as needed | Use 10 cc syringe and umbilical vein |  | 2 |  |
| Assess for and recognize sign(s) of placental separation | Gush of blood, cord lengthening,  Modified Brandt-Andrews |  | 1 |  |
| Deliver placenta | Controlled cord traction  Use correct vectors  Give suprapubic support to prevent prolapse |  | 3 |  |
| Ensure firm fundus at expected height |  |  | 2 |  |
| Manage excessive bleeding | Determine reason for bleeding  Fundal massage  Empty bladder  Provide correct order for at least one medication  Breastfeeding asap (as reasonable) |  | 5 |  |
| Check for and begin repair of lacerations | Request local anesthetic and suture  Inject local  Sew anchor stitch and tie knot |  | 5 | *Verbalize this during the OSCE. You wont have to actually do it at that time.  *During lab on day 1—make sure to demonstrate how to do a hand tie and instrument tie. |
| Examine placenta | Grossly normal size/shape/structure  Intact  3 vessel cord |  | 2 |  |
| Ensure patient comfort and recovery | Clean gown, blankets  Initiate breastfeeding  Briefly discuss perineal care: ice, peri bottle, sitz bath |  | 3 |  |
| Write delivery note | Report succinctly on all procedures above |  | 2 | *Please upload |
| IP OSCE Grade | Subtract 1 point per minute over 20 minutes, |  | 50 |  |

**Instructions for OSCE**

1. Prior to doing the check off please review the chart and EFM strip that will be available on canvas. You will then have 20 minutes to demonstrate proper management of the 2^nd^ and 3^rd^ stages of labor for that patient. Afterwards, you will write a delivery note, which must be uploaded to Canvas following completion of the OSCE.
2. The patient might possibly have some abnormal findings, i.e., preeclampsia, anemia, excessive bleeding at the time of delivery, etc.
3. One point will be subtracted for each minute beyond the 20 minute time limit. You may bring charting templates for the delivery note to the OSCE.
4. You are not allowed to bring any cheat sheets in for the OSCE.
5. The midwife should not talk to the faculty who is observing the OSCE unless the faculty is acting as patient. All discussion should be between the midwife and the pregnant woman—just like in real life. Try to provide information and teaching to the pregnant woman in a realistic way. For example, explain your findings as you do your exam.
