# Supplementary material for: Advancing Nurse‐Midwifery Education: A Quality Improvement Initiative for Competency‐Based Intrapartum Skills Laboratories
Source: J Midwifery Womens Health. 2025 Sep 20;71(2):283–9. doi: 10.1111/jmwh.70029 (PMC13067923; doi:10.1111/jmwh.70029)
Supplement: Supplementary file 4 — Table S4. Post‐laboratory Student Assessment Survey [file JMWH-71-283-s003.docx]

Table S4. Post-lab Student Assessment Survey

This survey is to assess your comfort level and confidence post-skills intensive. The survey is required as a part of the ongoing quality improvement initiative in the SON. It will ask your name, but only for the purpose of matching pre and post lab assessment. Once these are matched, the data will be de-identified. All questions on based on previous surveys and the NLN Student Satisfaction and Self-Confidence in Learning scale which is a a 1-5 Likert with 1 being strongly disagree and 5 being strongly agree. Thank you for your time in completing this brief assessment.

* Required

Student Name *

Your answer

I feel confident, post-lab, about the skills that were reviewed. *

Strongly Disagree

1

2

3

4

5

Strong Agree

I feel that simulation and practice time in the lab benefitted my learning. *

Strongly Disagree

1

2

3

4

5

Strongly Agree

I feel that simulation and lab time was well suited to my learning style. *

Strongly Disagree

1

2

3

4

5

Strongly Agree

I am confident that my faculty were well prepared to teach me skills necessary for midwifery practice. *

Strongly Disagree

1

2

3

4

5

Strongly Agree

The simulation provided me with a variety of learning materials and activities to promote my learning the midwifery curriculum. *

Strongly Disagree

1

2

3

4

5

Strongly Agree

I am confident that I am mastering the content of the simulation and lab activities that my instructors presented to me. *

Strongly Disagree

1

2

3

4

5

Strongly Agree

I am confident that I am developing the skills and obtaining the required knowledge from this simulation/lab intensive to perform necessary tasks in a clinical setting. *

Strongly Disagree

1

2

3

4

5

Strongly Agree

My instructors used helpful resources to teach the simulation/lab intensive. *

Strongly Disagree

1

2

3

4

5

Strongly Agree

I know how to use simulation and lab activities to learn critical aspects of these skills. *

Strongly Disagree

1

2

3

4

5

Strongly Agree

I know how to get help when I do not understand the concepts covered in the simulation/lab. *

Strongly Disagree

1

2

3

4

5

Strongly Agree

What additional content/skills would you find beneficial to incorporate into the lab? *

What can faculty do differently to improve your intrapartum lab intensives experience?
