# Supplementary material for: Advancing Nurse‐Midwifery Education: A Quality Improvement Initiative for Competency‐Based Intrapartum Skills Laboratories
Source: J Midwifery Womens Health. 2025 Sep 20;71(2):283–9. doi: 10.1111/jmwh.70029 (PMC13067923; doi:10.1111/jmwh.70029)
Supplement: Supplementary file 5 — Table S5. Pre‐Laboratory Intensive Assessment [file JMWH-71-283-s007.docx]

**Table S5. Pre-Lab Intensive Assessment**

This survey is to assess your comfort level and confidence in your midwifery skills to date. The survey is required as a part of the ongoing quality improvement initiative in the SON. It will ask your name, but only for the purpose of matching pre and post lab assessment. Once these are matched, the data will be de-identified. All questions on based on previous surveys and the NLN Student Satisfaction and Self-Confidence in Learning scale which is a 1-5 Likert with 1 being strongly disagree and 5 being strongly agree. Thank you for your time in completing this brief assessment.

Name:

|  | Strongly Agree | Agree | Neutral | Disagree | Strongly Disagree |
| --- | --- | --- | --- | --- | --- |
| I feel confident, pre-lab, about the skills that will be reviewed. |  |  |  |  |  |
| I feel that simulation and practice time in the lab will benefit my learning. |  |  |  |  |  |
| I feel that simulation and lab time is well suited to my learning style. |  |  |  |  |  |
| I am confident that my faculty are well prepared to teach me skills necessary for midwifery practice. |  |  |  |  |  |

Additional question:

What skills would you find beneficial to include in the lab?
