# Supplementary material for: Advancing Nurse‐Midwifery Education: A Quality Improvement Initiative for Competency‐Based Intrapartum Skills Laboratories
Source: J Midwifery Womens Health. 2025 Sep 20;71(2):283–9. doi: 10.1111/jmwh.70029 (PMC13067923; doi:10.1111/jmwh.70029)
Supplement: Supplementary file 6 — Table S6. Post‐Laboratory Intensive Assessment [file JMWH-71-283-s006.docx]

**Table S6. Post-Lab Intensive Assessment**

This survey is to assess your comfort level and confidence in your midwifery skills to date. The survey is required as a part of the ongoing quality improvement initiative in the SON. It will ask your name, but only for the purpose of matching pre and post lab assessment. Once these are matched, the data will be de-identified. All questions on based on previous surveys and the NLN Student Satisfaction and Self-Confidence in Learning scale which is a 1-5 Likert with 1 being strongly disagree and 5 being strongly agree. Thank you for your time in completing this brief assessment.

Name:

|  | Strongly Agree | Agree | Neutral | Disagree | Strongly Disagree |
| --- | --- | --- | --- | --- | --- |
| I feel confident, post-lab, about the skills that were reviewed. |  |  |  |  |  |
| I feel that simulation and practice time in the lab benefitted my learning. |  |  |  |  |  |
| I feel that simulation and lab time was well suited to my learning style. |  |  |  |  |  |
| I am confident that my faculty were well prepared to teach me skills necessary for midwifery practice. |  |  |  |  |  |
| The simulation provided me with a variety of learning materials and activities to promote my learning the midwifery curriculum. |  |  |  |  |  |
| I am confident that I am mastering the content of the simulation and lab activities that my instructors presented to me. |  |  |  |  |  |
| I am confident that I am developing the skills and obtaining the required knowledge from this simulation/lab intensive to perform necessary tasks in a clinical setting. |  |  |  |  |  |
| My instructors used helpful resources to teach the simulation/lab intensive. |  |  |  |  |  |
| I know how to use simulation and lab activities to learn critical aspects of these skills. |  |  |  |  |  |
| I know how to get help when I do not understand the concepts covered in the simulation/lab. |  |  |  |  |  |

Additional questions:

What additional content/skills would you find beneficial to incorporate into the lab?

What can faculty do differently to improve your intrapartum lab intensives experience?
