# Supplementary material for: Advancing Nurse‐Midwifery Education: A Quality Improvement Initiative for Competency‐Based Intrapartum Skills Laboratories
Source: J Midwifery Womens Health. 2025 Sep 20;71(2):283–9. doi: 10.1111/jmwh.70029 (PMC13067923; doi:10.1111/jmwh.70029)
Supplement: Supplementary file 7 — Materials S1. Revised Standards for Quality Improvement Reporting Excellence (SQUIRE 2.0) September 15, 2015 [file JMWH-71-283-s002.docx]

**Revised Standards for Quality Improvement Reporting Excellence (SQUIRE 2.0) September 15, 2015**

| **Text Section and Item Name** | **Section or Item Description** |
| --- | --- |
| **Notes to authors** | - The SQUIRE guidelines provide a framework for reporting new knowledge about how to improve healthcare - The SQUIRE guidelines are intended for reports that describe [system](#_bookmark13) level work to improve the quality, safety, and value of healthcare, and used methods to establish that observed outcomes were due to the [intervention(s)](#_bookmark8). - A range of approaches exists for improving healthcare. SQUIRE may be adapted for reporting any of these. - Authors should consider every SQUIRE item, but it may be inappropriate or unnecessary to include every SQUIRE element in a particular manuscript. - The SQUIRE Glossary contains definitions of many of the key words in SQUIRE. - The Explanation and Elaboration document provides specific examples of well-written SQUIRE items, and an in-depth explanation of each item. - Please cite SQUIRE when it is used to write a manuscript. |
| **Title and Abstract** |  |
| **1. Title** | Indicate that the manuscript concerns an [initiative](#_bookmark6) to improve healthcare (broadly defined to include the quality, safety, effectiveness, patient- centeredness, timeliness, cost, efficiency, and equity of healthcare)  Advancing Nurse-Midwifery Education: A Quality Improvement Initiative for Competency-Based Intrapartum Skills Labs |
| **2. Abstract** | 1. Provide adequate information to aid in searching and indexing 2. Summarize all key information from various sections of the text using the abstract format of the intended publication or a structured summary such as: background, local [problem,](#_bookmark10) methods, interventions, results, conclusions   Introduction: Maternal morbidity and mortality rates in the United States and Georgia remain alarmingly high, exceeding those of many low-resource regions despite extensive interventions. Research highlights the role of competent midwifery care in addressing key contributing factors, such as limited healthcare access, insufficient prenatal care, and adverse Social Determinants of Health (SDoH). To expand the pool of qualified certified nurse-midwives (CNMs), there is a pressing need for robust midwifery education programs, reliable and valid evaluation tools for student assessment, and documentation of skill development and confidence improvement among trainees.  Process: To evaluate preparedness and competency, a quality improvement project was initiated to assess the effectiveness and efficiency of the intrapartum simulation lab. A modified version of the National League for Nursing (NLN) Student Satisfaction and Self-Confidence in Learning tool was utilized. Third-semester nurse-midwifery students completed pre- and post-lab surveys, which included a Likert scale to measure confidence in the simulation lab’s ability to meet their educational needs. Qualitative questions were incorporated to identify suggestions for lab improvements.  Outcomes: Statistically significant improvements were observed in midwifery students’ pre- and post-lab assessments, particularly in their confidence regarding the skills reviewed, the alignment of simulation and lab time with their learning styles, and their trust in faculty members’ ability to effectively teach essential midwifery practices.  Discussion: The findings validate the effectiveness of intentional teaching strategies and innovative simulation technologies in enhancing midwifery education. Increasing the number of competent midwives in practice represents a critical step in addressing the persistently high maternal morbidity and mortality rates in the United States. These teaching approaches and technologies can also be applied to other midwifery labs within the program and adapted for use in other Advanced Practice Registered Nursing (APRN) specialties. |
| **Introduction** | *Why did you start?* |
| [**3. Problem**](#_bookmark10) [**Description**](#_bookmark10) | Nature and significance of the local [problem](#_bookmark10)  The intrapartum lab is critical for preparing midwifery students for clinical experiences by bridging theoretical knowledge and hands-on practice. However, improvements are needed to address identified gaps and enhance its effectiveness. Incorporating valid evaluation tools, structured simulation briefings, comprehensive feedback mechanisms, and additional faculty resources could improve student outcomes. |
| **4. Available knowledge** | Summary of what is currently known about the [problem,](#_bookmark10) including relevant previous studies.  While most scholars agree that competency is a combination of theoretical knowledge, practical skills, and the demonstration of specific behaviors^3,8,9,11,12^, measuring competency remains an elusive challenge^,3, 11^.  Many scholars agree on the need for reliable and valid tools to assess student competency^3,9,11,12,17^. Some suggest that standardized assessment tools could help measure competency across various programs^11,12,17^. Woeber further clarifies that student progression and preparedness are often not directly linked to the intended outcomes of learning or clinical competencies, pointing to a gap in accurate assessments^11^. In light of limited clinical sites and diverse educational needs, simulation-based learning has become an integral component of both pre-licensure and graduate nursing education.  Simulation provides a safe learning space where students can make mistakes, reflect on their experiences, and integrate their clinical skills^3,10,17^. |

| **5.** [**Rationale**](#_bookmark12) | Informal or formal frameworks, models, concepts, and/or [theories](#_bookmark14) used to explain the [problem,](#_bookmark10) any reasons or [assumptions](#_bookmark0) that were used to develop the [intervention(s),](#_bookmark8) and reasons why the [intervention(s)](#_bookmark8) was expected to work  The Knowledge to Action (KTA) framework has been successfully applied to nursing education^22^ and can be adapted to incorporate competency integration into midwifery curricula. Dolansky et al. proposed a three-step approach to KTA: assessing readiness, implementing small iterative changes through Plan-Do-Study-Act (PDSA) cycles, and developing an organized plan with clear goals, timelines, and milestones^22^ In addition, Alternative or Authentic Assessment models also offer a creative and individualized approach to evaluating student learning. This assessment method emphasizes practical demonstrations of knowledge and skills, often through nontraditional means. Authentic assessment recognizes that learning is a nonlinear process, and it values individualized growth, skill demonstration, and critical thinking over simply acquiring content knowledge ^26, 27^ |
| --- | --- |
| **6. Specific aims** | Purpose of the project and of this report  This quality improvement project aimed to evaluate the effectiveness and efficiency of an intrapartum lab after integrating evidence-based teaching strategies, innovative technology use, and assessments of midwifery student confidence both before and after the lab, alongside summative measures of student competency |
| **Methods** | *What did you do?* |
| **7.** [**Context**](#_bookmark1) | Contextual elements considered important at the outset of introducing the [intervention(s)](#_bookmark8)  The study took place in a large academic health center school of nursing within the nurse midwifery MSN program and involved 18 third-semester nurse-midwifery students, most of whom entered the program directly after completing an accelerated pre-licensure nursing program. Students came from diverse academic backgrounds, with most holding degrees in social or biological sciences and a range of life and work experiences. The faculty team consisted of four experienced nurse-midwives, including the program director, course coordinator, and lab coordinator, along with guest presenters from a nearby birth center and a doula. Three lab staff members provided technological support. |
| **8.** [**Intervention(s)**](#_bookmark8) | 1. Description of the [intervention(s)](#_bookmark8) in sufficient detail that others could reproduce it 2. Specifics of the team involved in the work   Initially, content assimilation was not formally assessed over the first four days of the lab, although individual feedback was provided as students reviewed material, refined their skills, and engaged in various simulations. Two summative evaluations were implemented: an online labor quiz (Appendix 1) administered before the lab intensives, and a mid-fidelity birth Objective Structured Clinical Examination (OSCE) (Appendix 2) conducted on the fourth day. Students scoring below 90% on either the quiz or OSCE were provided with remediation and additional faculty support, followed by a reevaluation the following week.  Pre-lab mini lectures were delivered to review key didactic content and set clear performance expectations for each session. Pre-lab preparation materials were enhanced to target information crucial for student success, and faculty resources were increased to provide clearer, more robust feedback. Opportunities for faculty-led skill demonstrations, as well as simulation pre-briefs and debriefs, were expanded. During the lab, enhanced simulation scenarios were employed, alongside increased structure and technology for skills practice. Planned experiences included amniotomy, internal monitor placement, suturing with realistic materials, and both mid- and high-fidelity simulations. The assessment component was redesigned to improve the OSCE, with faculty pre-lab training to ensure evaluation consistency. Students were required to complete self-reflective notes, along with pre- and post-lab surveys on content, confidence, satisfaction, and faculty proficiency. |
| **9. Study of the Intervention(s)** | 1. Approach chosen for assessing the impact of the [intervention(s)](#_bookmark8) 2. Approach used to establish whether the observed outcomes were due to the [intervention(s)](#_bookmark8)   Student surveys included a modified version of a validated National League for Nursing (NLN) tool to assess student confidence and satisfaction (Figure 1 and 2). The study utilized an observational, one-group posttest design to document outcomes following the four-day lab intensive with all 18 third-semester midwifery students. A pre-posttest student self-evaluation was administered via a five-question Likert scale, with an additional qualitative question regarding suggested lab improvements. The final component of the evaluation involved a one-group posttest design with skills checklists for the OSCE summative evaluation on the fourth day. Pre- and posttest surveys were matched and de-identified to ensure participant anonymity.  The effectiveness of the enhanced teaching strategies for the lab intensives was assessed through daily faculty observations, debrief discussions following each lab day, and student surveys. After each lab day, faculty gathered for debriefing and to confirm plans for the following day. A comprehensive faculty debrief took place two weeks after the lab, which included all faculty and lab staff, followed by a final debrief a month later to discuss the results and initial plans for future lab iterations. |
| **10. Measures** | 1. Measures chosen for studying [processes](#_bookmark11) and outcomes of the [intervention(s)](#_bookmark8), including rationale for choosing them, their operational definitions, and their validity and reliability 2. Description of the approach to the ongoing assessment of contextual elements that contributed to the success, failure, efficiency, and cost 3. Methods employed for assessing completeness and accuracy of data   Student surveys included a modified version of a validated National League for Nursing (NLN) tool to assess student confidence and satisfaction. The study utilized an observational, one-group posttest design to document outcomes following the four-day lab intensive with all 18 third-semester midwifery students. A pre-posttest student self-evaluation was administered via a five-question Likert scale, with an additional qualitative question regarding suggested lab improvements. The final component of the evaluation involved a one-group posttest design with skills checklists for the OSCE summative evaluation on the fourth day. Pre- and posttest surveys were matched and de-identified to ensure participant anonymity. |
| **11. Analysis** | 1. Qualitative and quantitative methods used to draw [inferences](#_bookmark5) from the data 2. Methods for understanding variation within the data, including the effects of time as a variable   Chi-square tests were conducted to assess whether a significant relationship existed between pre- and posttest survey results regarding students' confidence and satisfaction with the midwifery intrapartum lab intensive. |
| **12. Ethical**  **Considerations** | [Ethical aspects](#_bookmark2) of implementing and studying the [intervention(s)](#_bookmark8) and how they were addressed, including, but not limited to, formal ethics review and potential conflict(s) of interest  None noted. This QI project was submitted to organizational IRB and found not be research and was classified as quality improvement. There was not ethics review nor potential conflict of interest. |
| **Results** | *What did you find?* |
| **13. Results** | 1. Initial steps of the [intervention(s)](#_bookmark8) and their evolution over time (*e.g.*, time-line diagram, flow chart, or table), including modifications made to the intervention during the project 2. Details of the [process](#_bookmark11) measures and outcome 3. Contextual elements that interacted with the [intervention(s)](#_bookmark8) 4. Observed associations between outcomes, interventions, and relevant contextual elements 5. Unintended consequences such as unexpected benefits, problems, failures, or costs associated with the [intervention(s).](#_bookmark8) 6. Details about missing data   Statistically significant improvements were observed in students’ confidence levels concerning the skills covered during the lab. Students reported that simulation and practice time were beneficial to their learning, that the lab environment was well-suited to their learning styles, and that faculty were effectively equipped to teach the necessary skills for midwifery practice.  The means for six questions, which appeared only on the posttest, scored between 4.5 and 5 on a five-point Likert scale. These questions were:   1. The simulation provided me with a variety of learning materials and activities to promote my learning of the midwifery curriculum. 2. I am confident that I am mastering the content of the simulation and lab activities that my instructors presented to me. 3. I am confident that I am developing the skills and obtaining the required knowledge from this simulation/lab intensive to perform necessary tasks in a clinical setting. 4. My instructors used helpful resources to teach the simulation/lab intensive. 5. I know how to use simulation and lab activities to learn critical aspects of these skills. 6. I know how to get help when I do not understand the concepts covered in the simulation/lab.   The most frequent pretest responses (Table 2) indicated the skills students believed that suturing and hand maneuvers were the most critical skills for inclusion in the lab. Table 3 presents the most frequent posttest responses regarding additional content or skills that students felt would have been beneficial to include in the lab. Students indicated that more practice with normal birth from start to finish and exposure to various birth positions would have benefitted their learning and confidence.  When asked what faculty could do differently to improve their intrapartum lab intensive experience, students reported that the content on the first day was too slow, while the following days felt rushed (table 4). Students also made suggestions about restructuring supplementary content and increasing practice time with more opportunity for repetition of skills. |
| **Discussion** | *What does it mean?* |
| **14. Summary** | 1. Key findings, including relevance to the [rationale](#_bookmark12) and specific aims 2. Particular strengths of the project   This quality improvement (QI) project demonstrated that integrating evidence-based teaching strategies and technological innovations significantly enhanced nurse-midwifery student confidence and satisfaction with an intrapartum lab intensive. The student surveys conducted before and after the lab intensives provided statistically significant evidence that lab time was beneficial, matched students' learning styles, and that faculty were well-prepared to teach essential skills |

| **15. Interpretation** | 1. Nature of the association between the [intervention(s)](#_bookmark8) and the outcomes 2. Comparison of results with findings from other publications 3. Impact of the project on people and [systems](#_bookmark13) 4. Reasons for any differences between observed and anticipated outcomes, including the influence of [context](#_bookmark1) 5. Costs and strategic trade-offs, including [opportunity costs](#_bookmark9)   The findings align with previous studies that emphasize the value of simulation learning in improving student confidence, competence, critical thinking, decision-making, and the management of infrequent obstetric emergencies^3,10,17,18,19,20^.  The project measured substantial increases in students’ perceived knowledge acquisition and skill demonstration, both of which are key components of competency as identified by various nursing and midwifery experts^3,8, 9, 11,18^. Several of the new teaching strategies implemented in this lab were highlighted by Fullerton et al. as essential to competency-based education^3^. While the measurement of competency remains inconsistent, McMahon et al. and Woeber support the use of valid and reliable assessment tools to evaluate student competence^11,17^. This project utilized an adaptation of the proven and reliable Student Satisfaction and Confidence Survey, developed by the National League for Nursing (NLN)^34^. |
| --- | --- |
| **16. Limitations** | 1. Limits to the [generalizability](#_bookmark3) of the work 2. Factors that might have limited [internal validity](#_bookmark7) such as confounding, bias, or imprecision in the design, methods, measurement, or analysis 3. Efforts made to minimize and adjust for limitations   This project was limited by its small sample size and its focus solely on intrapartum content, which restricts the generalizability of the results. Future research could expand the sample size and explore a broader range of midwifery education content. Given the challenges in securing graduate-level clinical preceptors, the results from this project may be helpful in the recruitment and retention of preceptors, especially for students with limited labor and delivery experience. Demonstrating students' competency at the beginning of clinical experiences could improve preceptor confidence in mentoring these students. The goal moving forward is to integrate these effective teaching strategies and technological innovations into all future midwifery labs, while also exploring additional proven teaching, evaluation, and technological approaches. |
| **17. Conclusions** | 1. Usefulness of the work 2. Sustainability 3. Potential for spread to other [contexts](#_bookmark1) 4. Implications for practice and for further study in the field 5. Suggested next steps   This quality improvement (QI) project demonstrated the positive impact of evidence-based teaching strategies and technological innovations on nurse-midwifery students' confidence, satisfaction, and competency in an intrapartum lab intensive. The use of simulation and hands-on learning, coupled with pre- and post-lab assessments, significantly enhanced students' perceived knowledge acquisition and skill development. These findings align with previous research emphasizing the value of simulation in midwifery education, particularly in improving clinical skills and decision-making in obstetric emergencies.  Through faculty collaboration and thoughtful integration of feedback from prior student cohorts, this project addressed key areas for improvement in lab design, content delivery, and student assessment. Notable enhancements included increased simulation scenarios, more skill practice opportunities, and tailored instructional strategies. Additionally, the incorporation of the Knowledge to Action framework ensured that teaching strategies were continuously refined based on student input, promoting an adaptive and responsive learning environment.  Future research could expand the sample size and include a broader range of midwifery topics, with potential applications to other advanced practice registered nursing (APRN) programs. Additionally, findings from this project can inform future preceptor recruitment and retention efforts, particularly for students lacking prior labor and delivery experience. |
| **Other information** |  |
| **18. Funding** | Sources of funding that supported this work. Role, if any, of the funding organization in the design, implementation, interpretation, and reporting  None noted |
